# Barriers to outpatient education for medical students: a narrative review

**DOI:** 10.5116/ijme.5d76.32c5

**Published:** 2019-09-27

**Authors:** Ricardo Luiz Oliveira Franco, José Lúcio Martins Machado, Renato Satovschi Grinbaum, Gustavo José Martiniano Porfírio

**Affiliations:** 1 Instituto de Assistência Médica ao Servidor Público Estadual (IAMSPE), Pós Graduação em Ciências da Saúde, Brazil; 2 Universidade Municipal de São Caetano do Sul USCS, Curso de Medicina, Campus Bela Vista, Brazil

**Keywords:** Education, medical, undergraduate, ambulatory care, teaching methods, clinical clerkship, preceptorship

## Abstract

**Objectives:**

This study surveys medical education literature published over the last 25 years (1993–2018) to identify the factors scholars consider deleterious to outpatient teaching for medical students.

**Methods:**

This study conducts a review of medical education literature published between 1993 and 2018 using Medline, Lilacs, Ibecs, Cochrane Library, and Scielo databases. The following search terms were utilized: “Education, Medical, Undergraduate” AND “Ambulatory Care” AND “Teaching/methods” OR “Clinical Clerkship” OR “Preceptorship.” This study focuses on papers describing deleterious factors for outpatient teaching with medical students and analyzes their results, discussions, and conclusions sections.

**Results:**

Of the 363 articles obtained, this study selected 33 for analysis. These papers identify numerous factors as barriers to outpatient education. For didactic purposes, these factors are categorized into four barrier groups: environment-institution, academic staff, students, and patients. Academic staff-related teaching barrier was the most frequently mentioned obstacle. Intense care schedule with little teaching time was considered the most common and relevant barrier to outpatient medical education, followed by inappropriate teaching environment and inadequate supervision model.

**Conclusions:**

There is a lack of recent literature on studies focusing on barriers to effective outpatient medical education. Factors identified as harmful to outpatient education have been pointed out by course directors, academic staff, and students in the literature. However, many of these factors remain overlooked by educators, who can use these factors to modify their academic activities for more effective results.

## Introduction

The evolution of medicine has significantly influenced the development of medical education. In this regard, The Flexner Report, a study of medical education in Canada and the United States published in 1910, has influenced medical education curricula until recently. Abraham Flexner, an American researcher, proposed a new educational structure for medicine, prioritizing a hospital-centered model of education and teaching centered on academic staff.^1^ However, advances in medical practices over the last 25 years, particularly in the field of diagnostic and therapeutic resources, have resulted in a new and significant transformation in medicine. Indeed, there has been a progressive reduction in the number and length of hospitalizations as a result of these developments, as well as changes in the profiles of hospitalized patients. A greater presence of chronic patients with highly specialized and complex diseases has been observed in wards.^2^^,^^3^^,^^4^

Based on their observations of transformations in medical practices, several authors have argued that “clinical teaching in universities is migrating from wards to outpatient clinics.”^2^^,^^5^^,^^6^^,^^7^ For instance, in the 1990s, Young reported that, regardless of these changes, the traditional model of ward-centered education reflected a limitation of the educational focus and was insufficient for achieving a complete medical education.^6^ Thus, contrary to Flexner’s precepts, a strong argument that clinical teaching should be based on outpatient profiles began emerging among medical education scholars. Such research also mentions the models of basic and community-based care^.8,9 ^These arguments have impacted medical education. For example, several medical schools around the world progressively expanded the time allocated to outpatient clinics.^10^ Meanwhile, in 2001, the Accreditation Council of Graduate Medical Education (ACGME) stated that US medical students needed to spend at least a third of their total clinical experience in outpatient clinics. The ACGME’s Internal Medicine Requirements Program further noted that students needed to spend a period longer than 36 months, with at least one half-day period per week, in outpatient clinics.^2^

This migration to outpatient clinics has resulted in a new line of questioning, attracting the attention of scholars from various fields. Everything known about ward-centered medical education, including teaching strategies, case discussion methods and student assessment methods, have required review and reformulation in their application to outpatient clinics.^11^^,^^12^^,^^13^^,^^14^^,^^15^   In 2006, O’Neill asserted that “the outpatient clinic is the learning environment within a health facility, but it is also the place that poses the greatest barrier to learning.”^16^

Indeed, the teaching methods used in outpatient clinics are fundamentally different from those used in wards.^17^^,^^18^  In most internships in wards, groups of two or more students interview and examine patients who, in most cases, have already been diagnosed and their courses of treatment have already been established. According to the literature, these students see an average of two or maybe three patients in a morning or afternoon. Moreover, the majority of patients in wards have chronic and highly complex diseases, which is not always appropriate for the clinical level of students.^9^^,^^14^^,^^19^^,^^20^^,^^21^

In contrast, outpatient clinics offer more diverse and effective educational opportunities. In his classic study on the subject published in 1989, Wooliscroft has asserted that the outpatient clinic is the best, if not only, place for effectively training students in disease management since it allows students the chance to encounter a wide range of pathologies in several evolutionary phases.^13^ Academic staff have greater teaching opportunities, with the potential to address topics of medical ethics and preventive medicine. Students can acquire a greater understanding of the impact of diseases on patients and their families. Moreover, the clinical reasoning process is different in this practice scenario. Medical education in outpatient clinics also provides training in communication skills while prioritizing the medical-patient relationship.^13^ Outpatient clinics provide better venues for learning about the functioning of the health system and its costs, allowing students to develop certain medical skills.^13^ Consequently, students feel more stimulated and confident and enjoy better relationships with the patients and academic staff.^14^ Contact with a greater number of patients in this type of teaching is more favorable for student learning.^21^

However, despite such advantages, teaching in outpatient clinics also faces several educational problems. First, this teaching modality is still characterized by short, discontinuous, highly varied, and unpredictable interventions compared to teaching in wards.^11^^,^^22^^,^^23^^,^^24^^,^^25^^,^^26^ In outpatient clinics, students have fewer opportunities to directly observe patients and teaching results are more likely to be lost. Work typically focuses on patient management, and the student may end up observing more than doing.^17^ Notably, regardless of the benefits of outpatient teaching, no significant differences in knowledge obtained by students taught in wards and outpatient clinics have been observed.^21^

Despite the increase in outpatient teaching in medical schools in recent years, no current and specific studies have addressed the factors negatively impacting the quality of teaching in academic outpatient clinics. Indeed, the only two other studies addressing similar subjects were published more than 25 years ago. Published in 1989, Wooliscroft’s study argues that the most significant barriers to outpatient medical education are the costs of maintenance and financing of academic outpatient clinics, as well as patients accepting the presence of apprentices in the healthcare environment. Wooliscroft also noted the difficulty of hiring and maintaining qualified professionals for teaching and the consequences of this on medical gains and productivity.^13^ Published in 1993, Krackov’s study used different classifications for the so-called barriers to outpatient education. Based on his experience in academic outpatient clinics in five American universities, Krackov proposed a division of the barriers into three major categories: namely, institutional policy-related barriers, administrative issues, as well as academic and curricular issues. Krackov did not attribute weight to the barriers but believed them to be specific to each institution. He also noted the cost and lack of incentives for academic staff as significant barriers to outpatient education.^14^

A substantial change in the health care landscape over the last decade has influenced medical educational, creating some difficulties and doubts among educators involved with outpatient activities. However, as noted, more recent scholarship has not focused on issues negatively impacting medical education in outpatient clinics. Indeed, the majority of studies that describe such barriers are primarily focused on education strategies, student assessment or other issues related to outpatient education. As such, information is scarce and only describes occasional problems encountered during academic activity. Addressing this concern, this study reviews the medical education literature published between 1993 and 2018 to determine which factors are currently considered as barriers to outpatient teaching and academic performance by the students, educators, and principals of medical institutions. 

## Method

A narrative review of the literature published over the last 25 years, specifically 1993–2018, was conducted with the objective of extracting and synthesizing information related to the factors negatively impacting medical outpatient teaching for medical students. Ethical approval was not necessary because this study is a narrative review of the literature and does not involve primary data, such as data collection or analyses of individual patients.

### Identification and selection of articles

A search of publications was conducted using the Biblioteca Virtual em Saúde (BVSalud) platform, which provides access to Medline, Lilacs, Ibecs, Cochrane Library, and Scielo databases. The following MeSH descriptors and respective Boolean operators were used to search for studies: “Education, Medical, Undergraduate” AND “Ambulatory Care” AND “Teaching/methods” OR “Clinical Clerkship” OR “Preceptorship.” The terms “barrier” or “barriers” were not used in the search because they are not related to medical education and have not generated results related to the subject of this study.

This study selected articles in English, Portuguese and Spanish published between 1993 and 2018, describing outpatient academic activities with medical students. This study based its selection on the author(s) describing any factor that had negatively impacted or exacerbated outpatient medical education for medical students in the article. The following exclusion criteria were used: lack of participation of medical students in the research or lack of information regarding education for medical students, absence of activities related to outpatient care, and omission of records or descriptions of any negative factors associated with outpatient medical education.

**Figure 1:**
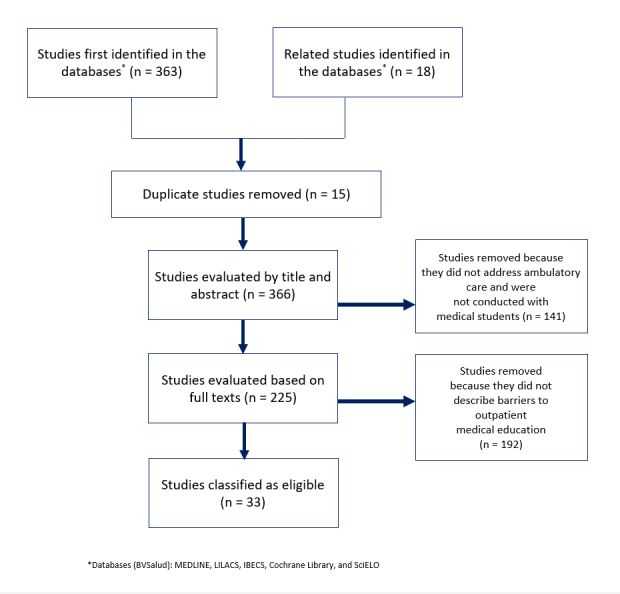
Flowchart showing the identification, screening, and selection stages in reviewing studies on barriers to outpatient medical education

The studies identified via this search process were inserted into a standardized table for data extraction and analysis, and duplicate studies were removed. Titles and abstracts were analyzed, and studies that were not conducted with medical students and outpatient activities were removed during the preliminary stage. Following this first selection, the results, discussions, and conclusions of the selected articles were read and analyzed in their entirety in order to identify descriptions of so-called “teaching barriers.” Figure 1 shows the process through which articles were identified and selected for the study.

### Data synthesis and analysis

Information extracted from the articles included the year of publication, type of publication, the methodology used, number of participants and description of barriers encountered. This information was independently reviewed by two of this study’s authors and subsequently by a third. The data obtained were grouped into common factors: namely, primary relationship with students, academic staff, patients or institutions. All differences of opinion were discussed and agreed upon by this study’s authors. The identified articles, as well as their thematic commonalities and characteristics are discussed in the next section.

## Results

The initial article search using the BVSalud platform generated 363 studies. Another 18 studies on related themes were found on the same platform or in references cited in the respective studies and added to the identified articles, for a total of 381 studies. Fifteen duplicate studies were then removed. The titles and abstracts of the remaining 366 studies were examined, and 141 studies were excluded because they were not conducted with medical undergraduates participating in outpatient care. The remaining 225 studies were analyzed in their entirety. After applying inclusion and exclusion criteria, 192 studies were classified as ineligible for this study as they did not describe any barriers to outpatient education faced by medical students. Ultimately, 33 articles were classified as eligible for this study.

### Study characteristics

Of the 33 articles classified as eligible for this study, 15 were categorized as research articles, 12 were categorized as either guidelines or expert opinions, and 2 were categorized as literature reviews. Of the four remaining articles, one article was categorized as medical conference material, one article provided a description of experiences in an academic outpatient clinic, one article presented a description of a program for hiring academic staff, and one article analyzed interviews with faculty members.

Of the studies categorized as research articles, 11 studies used information obtained from questionnaires completed by students after the outpatient stage, 6 studies used information obtained from questionnaires distributed to academic staff, and 2 used information obtained from questionnaires distributed to patients. One study used information obtained from a questionnaire completed by course directors, while another used information obtained from the analysis of clinical case presentation audios. A general description of the 33 studies classified as eligible, as well as the main barriers described can be found in the Appendix.

The analysis of these 33 studies classified as eligible generated a large amount of information. For didactic purposes, teaching barriers were divided into four large categories according to the main factor to which they relate: namely, barriers related to educational environments and institutions, academic staff-related barriers, patient-related barriers and student-related barriers. Table 1 describes the main barriers to outpatient medical education according to these categories.

### Educational barriers related to educational environments and institutions

Of the eligible studies 16 described barriers related to educational environments and institutions. Teaching environment significantly influences student learning.^6^ Academic outpatient clinics, where students and resident physicians undergo supervised training, are characterized by increasing maintenance costs and waiting time for patients.^2^ Two studies indicated the need to prepare the teaching environment for students.^19^^,^^31^ Several problems related to the outpatient teaching environment were described in the literature in view of the growing popularity of this practice scenario in recent years. When the scope of the health institution housing the outpatient clinic is focused on care rather than medical education, conflicts may emerge and negatively impact the quality of teaching. This fact was regarded as a significant problem by Israeli authors, who reported a lack of commitment by the government to maintaining the same quality of care in medical education.^27^

Some questions are directly related to the actions or inactions of the institutions. The failure to designate a professional dedicated to teaching in health institutions, the lack of strict criteria for hiring teaching professionals, and inadequate benefits for teaching are noted as important barriers to outpatient medical education.^14^^,^^28^^,^^29^ Other problems are more related to governmental spheres, such as the poor distribution of doctors across territories of countries. The most significant problems in this regard were reported by Australian authors, who found that the country’s geographical vastness and many scattered settlements hinder the localization of professionals by the government.^28^

Until the fourth year of undergraduate studies, most international medical schools focus on the so-called basic specialties, such as medical clinics, pediatrics, general surgery, gynecology and obstetrics, and collective health.^10^ Swiss authors have reported a shortage of primary care physicians, resulting in students primarily learning from specialists, which appears inadequate.^11^

North American authors tended to mention problems related to the maintenance costs of outpatient clinics, as well as the difficulties in hiring doctors from rural communities located far from universities to act as academic staff.^14^^,^^21^

**Table 1:** Main barriers to outpatient medical education

Main barriers	Evidences from literature
Barriers related to educational environments and institutions	Failure to recognize the need for excellence in teaching.^10,14,27,39^
	A lack of institutional support.^10,14,27,39^
	Conflicts between medical education and healthcare.^10,14,27,28,39^
	No contact/lengthy distances between the outpatient clinic and university.^7,10,14,29,31^
	Outpatientand university curricula not being integrated.^7,10,14,29,31^
	Academic costs/insufficient funding for academic outpatient clinics.^8,14,27,28,29,32,37^
	Inadequate financial incentives for academic staff.^10,14,27,28,30,32,37^
	Difficulties in the hiring of qualified academic staff.^10,14,27,28,29,30,32,37,38^ Inadequate criteria for the hiring of academic staff.^10^
	Absence of a professional dedicated to teaching at the institution.^32^
	Poor distribution of doctors.^7,8,11,17,28,29^
	Inappropriate or small care rooms.^2,5,6,10,11,14,17,18,21,23,24,27,28,31,32,34,40^
	Insufficient technological and audiovisual resources suitable for teaching.^32,35,36^
Academic staff-related	Intense and inadequate care schedule for teaching/insufficient time for teaching.^2,5,6,10,11,15,18,19,20,23,25,27,28,29,31,32,34,37,38,39,42^
	A lack of professional training and retraining.^31^
	Fear of losing private patients.^10,14,29,30^
	Fear of losing professional autonomy.^10,30^
	Inadequate supervision model/teaching method.^5,11,14,16,17,23,22,29,34,40^
	Inadequate service model for students.^11,20^
	Inappropriate or absence of feedback.^5,11,15,17,20,21,22,23,24,25,29,34^
Patient-related	The lack of suitable patients for teaching.^5,19,31,32^
	Failure to obtain patient consent for academic activities.^7,17^
	No follow-up/continuity of cases attended.^7,17^
	Absenteeism.^32^
Student-related	A lack of commitment to and interest in learning.^32^
	Increasing numbers of students in the internship group.^32,35,36^

These professionals do not see the benefits of keeping students in their services, and fear reduced earnings, a loss of patients, and the loss of professional autonomy.^10^^,^^14^^,^^30^ Moreover, the distance between universities and outpatient clinics means that the outpatient curriculum may not be integrated with that of the university. The integration of outpatient clinic and university curricula is considered an important motivating factor for learning, especially for beginner students in clinical courses.^10^^,^^31^   According to Karkabi, there is no established standard regarding what an academic outpatient clinic should contain in terms of equipment, rooms, and staff.^27^ Problems with the care environment, such as inadequate office space and furniture, a lack of equipment, and even the absence of an appropriate place for private discussions with students, were identified as the second most frequent set of barriers to outpatient medical education.^2^^,^^18^^,^^31^^,^^32^ Small offices unable to accommodate patients, faculty, and seated students were reported as a recurrent issue in several countries.^2^ Interestingly, studies found that, regardless of country, groups of students placed in a small office without sufficient chairs were accommodated on divans, embarrassing patients. Various studies recommend that the care environment be focused on teaching and, whenever possible, that at least two offices be available in academic outpatient clinics, with independent access, and a specific area for discussing cases far from patients.^2^^,^^10^^,^^17^^,^^19^^,^^28^^,^^31^^,^^33^^,^^34^

Several studies noted outpatient units without technological access to information as a significant factor negatively impacting the quality of education.^32^^,^^35^^,^^36^ Several studies advanced the need for access to information via the Internet due to its clear educational benefits. However, there was no consensus between studies regarding permission to use computers or other electronic devices during care insofar as they can take up more of a student’s time, as well as extend consultation and patient waiting times.^2^

### Academic staff-related teaching barriers

Of the eligible articles, 29 described academic staff-related barriers. Several authors reported the absence of an appropriate educational structure for teaching.^23^^,^^24^ Outpatient care is characterized by unpredictability, large numbers and case variability. Thus, it is a place naturally exposed to service pressures.^11^^,^^23^^,^^31^ Insufficient time for teaching, a factor often related to academic staff, as well as the requirements of health institutions, especially those with high patient demands, was found to be the most frequent and relevant barrier to outpatient medical education in several studies.^2^^,^^5^^,^^6^^,^^10^^,^^11^^,^^15^^,^^18^^,^^19^^,^^20^^,^^23^^,^^25^^,^^27^^,^^28^^,^^29^^,^^31^^,^^32^^,^^34^^,^^37^^,^^38^^,^^39^^,^^42^ Moreover, one study noted that the diversity of diagnoses in outpatient clinics is a limiting factor for teaching, making it difficult to standardize the curriculum.^21^

The value of feedback for student learning is unquestionable.^11^^,^^20^Academic staff unfamiliar with this tool or how to use it or who did not make use of feedback in case discussions were common reasons for student complaints. The absence of feedback is considered an important barrier to outpatient medical education, primarily because of the proven and established educational benefits that this tool provides.^21^^,^^22^^,^^23^^,^^34^ According to the surveyed studies, short, non-specific or inappropriate feedback, or even the absence of feedback, was intrinsically related to the short time available for teaching.^15^^,^^21^^,^^22^^,^^25^^,^^38^^,^^40^

Using questionnaire data, four studies noted that students reported that the academic staff rarely discussed cases they attended, even when there was time available. These studies also found that students tended to remain passive during consultations, observing more than doing.^11^^,^^21^^,^^38^^,^^41^ Faculty members must undergo refresher courses and professional training in order to remain up to date.^10^^,^^31^ They need to have knowledge of case discussion techniques and adequate time to apply them.^11^^,^^20^ The choice of an inadequate care model or technique for discussing cases thus constitute a significant barrier to outpatient education^.11,20^ Discussions of cases using established models, such as SNAPPS and the One-Minute Preceptor, can be systematized for the cases of all attended patients, even in outpatient clinics with high demands. These models also allow for the cultivation of more active and participatory attitudes toward learning among students.^11^

### Patient- and student-related educational barriers

Of the eligible articles, 9 described student-related barriers and 7 described patient-related barriers. In addition to preparing the environment for the student, suitable patients must be chosen for the activity in accordance with the student’s level.^19^^,^^31^^,^^37^ Indeed, several studies note that it is a mistake to think that a student will learn in any practice setting and from any academic staff.^19^^,^^31^^,^^37^ 

Medical education scholars often divide students into groups based on their experience or course progress, such as “beginners in the clinical course” (third- and fourth-year medical students) and “advanced in the clinical course” (interns). “Beginners in the clinical course” suffer the most from inadequate environment preparation and patient choice.^35^ Students in this phase require greater supervision and interaction with patients whose diagnoses correlate with their university curriculum. Furthermore, student interns need greater autonomy under supervision and more demanding activities with randomized patients. Failure to do so will result in lower learning rates.^17^^,^^30^^,^^35^

Individual studies noted further student-related barriers. Some students reported via questionnaires that they felt uncomfortable with the possibility of harming patients as a result of increased waiting time. Although research indicates that most patients agree to participate in academic activities, it is important that they are aware that, in outpatient clinics, care is more time-consuming and requires longer waiting times.^33^ This understanding prevents significant stress for academic staff, students, and patients.^5^^,^^32^^,^^33^ One study noted several student-related barriers, including student difficulties in prescribing medications, as well as addressing language barriers and different beliefs of students and patients.^15^ Another study noted a lack of student interest and commitment as barriers to teaching.^32^ The same study reported that patient absenteeism is an important barrier to teaching, but did not suggest any solutions to mitigate this issue.^32^

Finally, the increasing number of students per internship group constitutes as an important barrier to outpatient medical education. Furthermore, few studies described an increase in the number of faculty members or their workload, indicating that these professionals usually continued fulfilling the same didactic obligations. In addition to changing the dynamics of care and case discussions, a higher number of students significantly compromises the ability of academic staff to provide feedback and evaluations.^15^^,^^32^^,^^35^^,^^36^

## Discussion

### Implications for medical education

Surveying medical education literature related to education barriers in outpatient clinics published between 1993 and 2018, this study identifies a considerable number of factors negatively influencing academic goals, including lack of institutional support, lack of preparation or adequate working conditions, and overly high number of students in internship groups. However, the reviewed studies consider many of these factors modifiable.

This study is aware that while different countries and regions face varying realities and problems, most of these problems are rooted in the service environment used for teaching students not being geared toward academic uses. In fact, according to some authors cited in this review, many institutions take advantage of an environment aimed at assisting the population, which is their primary objective, to develop academic activities. Arguably, one of the major problems of outpatient education is rooted in this behavior.

Based on this survey, this study proposes a fully structured and fully university-controlled environment for academic activities, where academic staff can determine their attendance schedule and choose the best patients according to their student’s level. Consequently, academic staff will have more contact with the university and its programs, as well as more time and better conditions in which to use appropriate teaching strategies, thereby providing better quality feedback and using more effective assessment methods.

### Study limitations

Although this narrative review was performed using a relevant, systematic, and easily replicable methodology, it has several limitations. The articles deemed eligible for this study were diverse in both nature and classification. Most studies discussed strategies or teaching methods used in outpatient clinics and were classified as qualitative research. The information obtained from these articles was based on questionnaires disseminated among academic staff, students or course directors and thus based on the opinions of the respondents and interviewees. None were specifically designed to determine barriers to ambulatory medical education or how these barriers influence the proposed educational objectives with scientific evidence. While information regarding the barriers was extracted from the results, discussion or conclusion sections of these studies, such information was usually limited to sub-items described in these articles. Given the heterogeneous nature of the extracted information, this study did not conduct qualitative analyses. While these limitations compromise the value of this study’s results, that the same negative factors are frequently mentioned in different studies by actors involved in outpatient medical education is noteworthy. It should also be emphasized that, for didactic purposes, this study classified barriers according to the most intrinsically related factors of origin. However, some barriers were related to more than one factor. For example, an intense care agenda was considered an academic staff-related barrier but is often an institutional imposition and not the choice of the professional. Similar reasoning may apply to lack of training/retraining courses or the chosen care model. Moreover, many of the teaching barriers indicated here are not exclusive to outpatient clinics. They can be found in other educational scenarios, including wards, day hospitals, and emergency rooms.

## Conclusions

This study surveyed the medical education literature published in the last 25 years for studies identifying barriers of outpatient medical education for students. While no current studies have specifically sought to determine these barriers, the literature does reveal a significant number of factors identified by students, academic staff, and course directors as deleterious to this teaching modality. These studies suggest that both human and structural components of outpatient clinic education may negatively influence medical teaching in various ways.

However, we believe that the determination of what is considered a barrier to outpatient medical education cannot be based solely on the opinion of the people involved. While there is much to learn about these teaching barriers, the factors identified in this study can be modified by educators and course directors in the organization and practice of their academic activities. Going forward, it is necessary to develop specifically designed and structured research projects to determine how these barriers influence the educational objectives proposed by universities, particularly through the use of combined medical competence assessment methods applied to students. Future studies that can more specifically determine what these barriers are and how much they alter the proposed educational objectives are required and will certainly benefit students, faculty staff, and institutions.

### Attributions of each researcher author

All authors were actively and substantively involved in the design and execution of this project. All authors participated in the identification of keywords and the search strategy. RLOF and JLMM independently read and analyzed the studies identified via the keyword search and participated in tabulating data for analysis and data extraction. All research authors discussed the focus of each study independently. All authors participated in the analysis and interpretation of data, as well as in the preparation of the final text. All authors have read and approved the final manuscript.

### Conflict of Interest

The authors declare that they have no conflict of interest.

## Supplementary material

Supplementary file 1 Appendix. General characterization of the studies classified as eligible in this research, published between 1993 and 2018
