# Supplementary material for: Barriers to outpatient education for medical students: a narrative review
Source: Int J Med Educ. 2019 Sep 27;10:180–90. doi: 10.5116/ijme.5d76.32c5 (PMC6773369; doi:10.5116/ijme.5d76.32c5)
Supplement: Supplementary file 1 — Appendix. General characterization of the studies classified as eligible in this research, published between 1993 and 2018 [file ijme-10-180-S1.pdf]

Appendix

General characterization of the studies classified as eligible in this research, published between 1993 and 2018

Author, Year	Publication type	Methodology	Main barriers described
Dent et al., 2001[36]	Research	Questionnaires applied to students (n = 86, response rate = 65%), teachers (n = 25, response rate 48%), and patients (n = 25, response rate = 100%)	Growing number of students in stage groups, as well as a lack of technical and audiovisual resources
Denton et al., 2015[29]	Report of conference material related to medical education	N/A	Inadequate supervision model, inadequate feedback, financial pressures on the institution, difficulty in hiring appropriate teachers, fear of patient loss by teachers, as well as long distances between ambulatory care and university
Gordon et al., 2000[28]	Guideline/Expert opinion	N/A	Poor distribution of doctors, short teaching time/intense patient agenda, difficulty hiring suitable teachers, lack of a suitable and structured teaching location, lack of adequate funding, and competitiveness of teaching with patient care
Dent, 2005[35]	Guideline/Expert opinion	N/A	Lack of audiovisual resources and technological access to information, as well as the growing number of students in stage groups
Schultz et al., 2004[37]	Research	Questionnaires applied to students (n = 532, response rate = 48%)	Short teaching time/intense patient agenda, difficulty in hiring appropriate teachers, and financial pressures from the service
Salerno et al., 2002[23]	Analysis of audiotapes	Analysis of audiotapes of interviews with preceptors (n =30, response rate = 30%)	Short teaching time/intense patient agenda, inadequate supervision model, inadequate feedback, as well as the lack of an adequate and structured place for teaching
Dent et al., 2003[19]	Guideline/Expert opinion	N/A	Inadequate patients for teaching, and intensive care agenda
Williams et al., 2013[8]	Literature review	N/A	Poor distribution of doctors and lack of adequate funding
Karkabi et al., 2010[27]	Guideline/Expert opinion	N/A	Short teaching time/intense patient agenda, difficulty in hiring appropriate teachers, financial pressures of the service, lack of an adequate and structured place for teaching, and a lack of concern for excellence in teaching by institutions
Leiva et al., 2012[32]	Research	Questionnaires applied to teachers (n = 6, response rate =100%)	Lack of a teacher dedicated to teaching, short teaching time/intense patient agenda, difficulty hiring suitable teachers, financial pressures of the service, lack of an adequate and structured teaching location, lack of audio-visual resources and technological access to information, inadequate patients for teaching, patient absenteeism, growing number of students in stage groups, as well as lack of student commitment to teaching
Bardgett et al., 2011[31]	Guideline/Expert opinion	N/A	Lack of training and refresher courses for teachers, short teaching time/intense patient agenda, lack of an adequate and structured place for teaching, long distances between the outpatient clinic and university, as well as inadequate patients for teaching

Myung et al., 2010[5]	Research	Questionnaires applied to students (n = 499, response rate = 100%)	Short teaching time/intense patient agenda, inadequate supervision model, inadequate feedback, lack of an adequate and structured place for teaching, as well as Inadequate patients for teaching
Wolpaw et al., 2009[11]	Research	Analysis of clinical case presentation audios (n = 215) between two groups of students: namely, those using and those not using SNAPPS technique. (Number of students, n = 162, response rate = 66,67%)	Poor distribution of doctors, short teaching time/intense patient agenda, inadequate supervision model, Inadequate feedback, as well as the lack of an adequate and structured place for teaching
Wooley et al., 2008[24]	Description of an academic outpatient model	N/A	Inadequate feedback, as well as the lack of a suitable and structured teaching site
Lake et al., 2006[17]	Guideline/Expert opinion	N/A	Poor distribution of physicians, inadequate supervision model, inadequate feedback, lack of an adequate and structured place for teaching, as well as discontinuity in patient follow-up
O'Neill et al., 2006[16]	Research	Questionnaires applied to teachers (n = 311, response rate = 62%)	Inadequate supervision model
Dolmans et al., 2002[22]	Research	Questionnaires applied to students (n = 1207, response rate = 82%)	Inadequate supervision model and feedback
Ferenchick et al., 2002[2]	Literature review	N/A	Short teaching time/intense patient agenda, as well as the lack of an adequate and structured place for teaching
Hunt et al., 1999[7]	Research	Questionnaires applied to course directors (n=112, response rate= 100%).	Discontinuity in patient follow-up, long distance between outpatient clinic and university, and poor distribution of physicians (specifically pediatricians)
O'Malley et al., 1999[34]	Research	Questionnaires applied to students (n = 24) and teachers (n = 14) Study includes analysis of 103 teaching sessions	Short teaching time/intense patient agenda, inadequate supervision model, inadequate feedback, as well as the lack of an adequate and structured place for teaching
McGee et al., 1997[20]	Guideline/Expert opinion	N/A	Short teaching time/intense patient agenda and inadequate feedback
Denton et al., 2015[30]	Description of teacher hiring program	N/A	Difficulties in hiring and retaining teacher doctors, insufficient teacher remuneration, and fear of patient loss by teachers
Fields et al., 2000[10]	Guideline/Expert opinion	N/A	Short teaching time/intense patient agenda, difficulties in recruiting and retaining teacher doctors, inadequate teacher salaries, fear of patient loss by teachers, lack of an adequate and structured place for teaching, as well as long distances between the outpatient clinic and university
Croft et al., 2012[18]	Research	Questionnaires applied to students (n = 44), teachers (n = 11), and patients (n = 44)	Short teaching time/intense patient agenda, as well as the lack of an adequate and structured place for teaching
Young et al., 1998[6]	Research	Questionnaires applied to students (n = 13)	Short teaching time/intense patient agenda, as well as the lack of an adequate and structured place for teaching
Hundertmark et al., 2018[21]	Research	Likert questionnaires applied to students in participant and control groups (n = 64); also includes literature review and expert opinion	Short teaching time/intense patient agenda and inadequate feedback

Dusch et al., 2018[38]	Research	Questionnaires applied to students (n = 75, response rate = 83%)	Short teaching time/intense patient agenda
Braverman et al., 2016[15]	Research	Questionnaires applied to students (n = 518)	Short teaching time/intense patient agenda and inadequate feedback
Bösner et al., 2017[25]	Research	Questionnaires applied to students (n = 36, response rate = 55,6%) and teachers (n = 35, response rate = 62,9%). Researchers also analyzed videotapes of attendances	Short teaching time/intense patient agenda and inadequate feedback
Serrao et al., 2016[42]	Guideline/Expert opinion	N/A	Short teaching time/intense patient agenda
Aluko et al., 2018[39]	Guideline/Expert opinion	N/A	Short teaching time/intense patient agenda, as well as conflicts between medical education and patient care
Krackov et al., 1993[14]	Guideline/Expert opinion	N/A	Institutional resistance to the financing of academic outpatient clinics, conflicts between medical education and patient care, inadequate teacher remuneration, fear of loss of productivity on the part of teachers, lack of an adequate and structured place for teaching, difficulty hiring suitable teachers, long distances between the outpatient clinic and university, as well as an inadequate supervision model
Masood et al., 2006[40]	Guideline/Expert opinion	N/A	Inadequate supervision model without academic Structuring
